# Super-Capacitive Performance of Manganese Dioxide/Graphene Nano-Walls Electrodes Deposited on Stainless Steel Current Collectors

**DOI:** 10.3390/ma12030483

**Published:** 2019-02-04

**Authors:** Roger Amade, Arevik Muyshegyan-Avetisyan, Joan Martí González, Angel Pérez del Pino, Eniko György, Esther Pascual, José Luís Andújar, Enric Bertran Serra

**Affiliations:** 1 ENPHOCAMAT (FEMAN) Group, Department of Applied Physics, Universitat de Barcelona, Martí i Franquès 1, E-08028 Barcelona, Spain; amusheghyan91@ub.edu (A.M.-A.); joanmarti13@gmail.com (J.M.G.); epascual@ub.edu (E.P.); jandujar@ub.edu (J.L.A.); ebertran@ub.edu (E.B.S.); 2 Institute of Nanoscience and Nanotechnology (IN2UB), Universitat de Barcelona, E-08028 Barcelona, Spain; 3 Instituto de Ciencia de Materiales de Barcelona, Consejo Superior de Investigaciones Científicas (ICMAB-CSIC), Campus UAB, E-08193 Bellaterra, Spain; aperez@icmab.es (A.P.d.P.); egyorgy@icmab.es (E.G.)

**Keywords:** inductively-coupled plasma, carbon nanostructures, electrochemical properties, thermal annealing

## Abstract

Graphene nano-walls (GNWs) are promising materials that can be used as an electrode in electrochemical devices. We have grown GNWs by inductively-coupled plasma-enhanced chemical vapor deposition on stainless steel (AISI304) substrate. In order to enhance the super-capacitive properties of the electrodes, we have deposited a thin layer of MnO_2_ by electrodeposition method. We studied the effect of annealing temperature on the electrochemical properties of the samples between 70 °C and 600 °C. Best performance for supercapacitor applications was obtained after annealing at 70 °C with a specific capacitance of 104 F·g^−1^ at 150 mV·s^−1^ and a cycling stability of more than 14k cycles with excellent coulombic efficiency and 73% capacitance retention. Electrochemical impedance spectroscopy, cyclic voltammetry, and galvanostatic charge/discharge measurements reveal fast proton diffusion (1.3 × 10^−13^ cm^2^·s^−1^) and surface redox reaction after annealing at 70 °C.

## 1. Introduction

Carbon-based nanostructured electrodes are suitable for different electrochemical applications due to their large surface area, thermal, and chemical stability, mechanical strength, and high conductivity. Carbon nanotubes (CNTs), graphene, and graphene nano-walls (GNWs) are promising materials to improve performance of different electrochemical devices such as sensors, batteries, electrochemical capacitors, and fuel cells [1,2,3,4,5,6].

Among the different carbon nanostructures, graphene nano-walls is one of the most recently developed and less investigated material. GNWs can be grown by different plasma enhanced chemical vapor deposition (PECVD) techniques such as microwave PECVD (MW-PECVD) [7], capacitively-coupled radio-frequency PECVD (CC-rf-PECVD) [8], direct current PECVD (DC-PECVD) [9], and inductively coupled plasma enhanced chemical vapor deposition (ICP-CVD) [10]. ICP-CVD is one of the high plasma density and large plasma volume techniques, which allow synthesizing GNWs at high deposition rates. In addition, ICP-CVD has a simple geometry and operates at relatively low pressures. There are two geometric designs for ICP reactor known as planar and cylindrical coil geometry [11]. The main method used in this work is the ICP-CVD method with a cylindrical coil geometry and substrate at floating potential inside the plasma.

ICP-CVD allows the growth of GNWs on different substrates without the need of a diffusion barrier nor a catalyst typically used for the growth of CNTs. Consequently, the synthesis process is greatly simplified. The growth mechanism of GNWs is still under discussion, but the first stage seems to be the deposition of an amorphous carbon layer on the substrate, which acts as a catalyst for the growth of the nano-walls [1].

Carbon-based nanostructured electrodes such as GNWs are especially promising for supercapacitor devices. These electrochemical devices present higher power density values than batteries as well as higher energy density values than electrolytic capacitors. Supercapacitors are used and are under development to improve the performance of electric vehicles, memory back-ups, and absorption of high peak power transients. Recently, several articles have shown the promising properties of GNWs as electrodes for electric double layer capacitors (EDLC). Researchers have been able to grow them on metallic substrates [12,13,14], flexible substrates [15], controlling their surface functionalization [16], their morphology [17], and obtaining high voltage energy storage devices [18]. However, very few articles have explored the electrochemical properties of metal oxide/GNWs nanocomposites for energy storage devices [19,20,21].

There are two main charging mechanisms in a super-capacitor such as electrostatic due to the formation of an electric double layer at the interface between the solid electrode and the ions in the electrolyte (double-layer capacitance), and through redox reactions near the surface region of transition metal oxides or conductive polymers involving electrolyte ions (pseudo-capacitance). In general, carbon electrodes have a higher contribution of double layer capacitance (DLC) than pseudo-capacitance (PC) [22]. Due to their rich redox behavior, thermal and chemical stability, transition metal oxides provide higher energy density than conventional carbon materials and better electrochemical stability than polymer materials [23].

Among super-capacitive transition metal oxides, manganese dioxide is one of the most investigated due to its environmental friendliness, cost, and low toxicity [23]. The proposed mechanism for pseudo-capacitance in manganese oxides is described by the following equation [24].

(1) $${\mathrm{MnO}}_{\alpha }{\left(\mathrm{OC}\right)}_{\beta }+{\delta C}^{+}+{\delta e}^{-}↔{\mathrm{MnO}}_{\alpha -\delta }{\left(\mathrm{OC}\right)}_{\beta +\delta }$$

where C^+^ are protons or alkali metal cations (Li^+^, Na^+^, K^+^) in the electrolyte. In the case of adsorption/desorption of protons, MnO_α_(OH)_β_ and MnO_α−δ_(OH)_β+δ_ indicate interfacial manganese hydroxides at high and low oxidation states, respectively. A major drawback of manganese dioxide is its low conductivity that reduces the charging/discharging rate of the electrode. Thus, a thin layer of the oxide is deposited to minimize the series resistance. In addition, a thermal treatment may help improve the contact between the transition metal oxide and the carbon-based nanostructured electrode improving the charge transfer process [25]. Optimization of this thermal treatment allows maximum charge/discharge rates without sacrificing electrochemical performance. 

Different methods can be used to deposit and prepare MnO_2_, such as hydrothermal [26], sol gel methods [24,27], and electrodeposition [24,27,28]. In previous studies, we have demonstrated the excellent electrochemical performance of galvanostatically electrodeposited MnO_2_ on vertically aligned carbon nanotubes (VACNTs) [28,29] as well as laser-coated MnO_2_-VACNTs [30]. In addition, there is a need to reduce overall costs of electrochemical devices by the use of cost-effective current collectors such as stainless-steel foil. This substrate has the advantages of being flexible, relatively chemically stable, and with low resistance.

In this case, we have grown GNWs on stainless steel AISI304 (SS) by the ICP-CVD method, which electrodeposited a thin layer of MnO_2_. Afterward, we have performed a thermal annealing process to improve the electrochemical properties of the electrodes and analyzed their performance. A mild thermal treatment at 70 °C resulted in ultrafast charge/discharge rates without giving up performance and stability.

## 2. Materials and Methods 

### 2.1. Growth of GNWs and MnO_2_/GNWs

**Figure 1:**
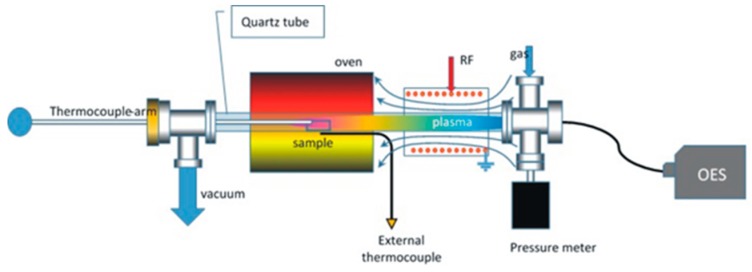
Schematic drawing of the ICP-CVD tubular reactor for producing carbon structures. The reactor is composed of a quartz tube, an oven, and a coil connected to an RF power supply. The sample is placed in the middle of the oven. The system is evacuated with a primary rotatory pump and the pressure were analyzed with a pressure meter. The temperature is measured by means of two thermocouples. Different gases can be introduced through an inlet at one end of the quartz tube. OES: Optical Emission Spectroscopy device to study the emission of plasma generated species.

Graphene nano-walls were synthesized on the SS substrate (100 μm thick and about 2 cm^2^ geometrical area) using an inductively coupled plasma chemical vapor deposition (ICP-CVD) (13.56 MHz, power 440 W) system [31] consisting on a long quartz tube (Vidrasa S.A., Ripollet, Spain) having a radio-frequency resonator (homemade), for producing remote plasma, and a tubular oven (PID Eng & Tech S.L., Madrid, Spain) working up to 1100 °C, 20 cm away from the quartz tube (Figure 1).

The sample was placed inside the quartz tubular reactor, which was evacuated down to a pressure below 1 Pa and heated up to 750 °C. Then, pure methane (99.995%) was introduced as a precursor gas at one end of the quartz tube (10 sccm) and the pressure was maintained in the range of 50 to 60 Pa. Under these conditions, plasma was ignited at an RF power of 440 W for 40 min. Lastly, the sample was cooled down to room temperature for 30 min. The areal density of the GNWs was measured with a microbalance and found to be about 58 μg·cm^−2^.

The electrochemical deposition was carried out using a galvanostatic method described elsewhere [28]. GNWs were used as anode and a graphite electrode as the cathode in a 0.2 M Na_2_SO_4_ aqueous solution with about 4 cm separation between electrodes. About 0.5 cm^3^ of a 0.2 M·MnSO_4_·H_2_O solution was added drop wise to the electrolyte through a hole in the cathode applying 1 mA cm^−2^ for 2 min [32]. After this process and previous to the heat treatment, the loading mass of MnO_2_ was determined by UV–vis spectrophotometry (PerkinElmer Inc., Shelton, CT, USA). First, MnO_2_ was dissolved in concentrated nitric acid. Then, manganese ions were oxidized to MnO_4_^−^ using K_2_S_2_O_8_ and AgNO_3_. From the characteristic absorbance maximum at 525 nm, the concentration of MnO_4_^−^ could be determined. From this value, the mass loading of MnO_2_ was calculated, which was about 16 μg [32] (see supplementary information, Figure S1).

The MnO_2_/GNWs samples were annealed at different temperatures to study their electrochemical performance: 70 °C, 200 °C, and 600 °C. The annealing process was carried out for 20 min under an argon atmosphere to avoid oxidation of the SS substrate. MnO_2_/GNWs without annealing (room temperature) and bare GNWs samples were also characterized for a comparison.

### 2.2. Samples Characterization

The microstructure and morphologies of the grown samples were investigated using field emission scanning electron microscopy (SEM) (JEOL JSM-7001F, operated at 20 kV, JEOL Ltd., Tokyo, Japan) and transmission electron microscopy (TEM) (JEOL 1010, operated at 200 kV, JEOL Ltd.). The carbon structures were transferred to the TEM grid by simply scratching off from the substrates. 

XPS (X-ray photoelectron spectroscopy) experiments were performed in a PHI 5500 Multi-Technique System (Physical Electronics, Chanhassen, MN, USA) with a monochromatic X-ray source (Aluminium Kalfa line of 1486.6 eV energy and 350 W), placed perpendicular to the analyzer axis, and calibrated using the 3d5/2 line of Ag with a fullwidth at half maximum (FWHM) of 0.8 eV. The analyzed area was a circle of 0.8 mm diameter, and the selected resolution for the spectra was 187.5 eV of pass energy and 0.8 eV/step for the general spectra and 11.75 eV of pass energy and 0.05 eV/step for the spectra of the different elements in the depth profile spectra. All measurements were made in an ultra-high vacuum (UHV) chamber pressure between 5 × 10^−9^ and 2 × 10^−8^ torr.

The physical and chemical characteristics of, as grown structures, were studied by Raman spectroscopy using a microscope HR800 (LabRam) (HORIBA France SAS, Palaiseau, France) with a 532 nm solid-state laser (5 mW laser power).

The electrochemical properties of the samples were analyzed by using cyclic voltammetry (CV), electrochemical impedance spectroscopy (EIS), and galvanostatic charge/discharge (GCD) cycling in a 1 M Na_2_SO_4_ aqueous solution using a potentiostat/galvanostat (AutoLab, PGSTAT30, Eco Chemie B.V., Utrecht, The Netherlands). All experiments were carried out at room temperature in a typical three-electrode cell. A Ag/AgCl electrode (3 M KCl internal solution) and a Pt-ring electrode were used as the reference and counter electrode, respectively. The working electrode was a sample of GNWs or MnO_2_/GNWs nanocomposite. The geometrical area of the working electrode was set to a constant value of 0.57 cm^2^.

The CV analysis was performed using a voltage window of 0.0 to 0.7 V vs. Ag/AgCl at scan rates between 10 and 150 mV·s^−1^. An alternating voltage of 10 mV amplitude was applied to the samples between 1 Hz and 100 kHz for the EIS analysis. Lastly, several thousands of charge/discharge cycles were applied to the samples between 0.7 V and 0.0 V vs. Ag/AgCl at different current densities.

## 3. Results

### 3.1. Morphological and Structural Characterization

**Figure 2:**
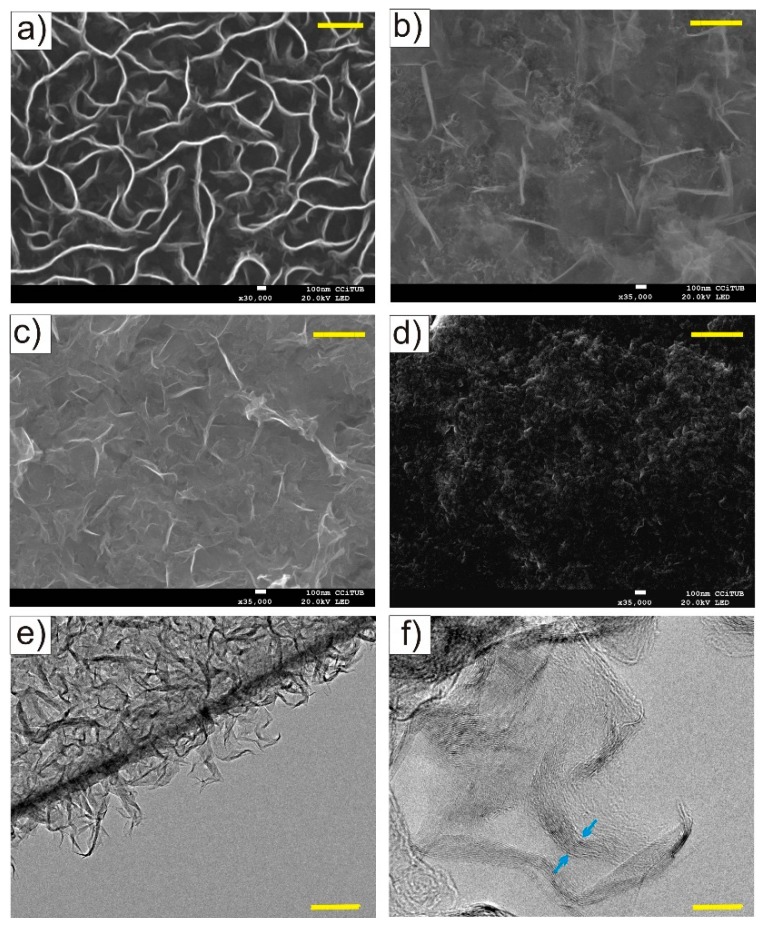
SEM images of GNWs grown on SS, (**a**) MnO_2_/GNWs without thermal treatment, (**b**) MnO_2_/GNWs with annealing at 200 °C (**c**) and 600 °C (**d**). TEM images of as grown GNWs (**e**), (**f**). Arrows indicate thickness of the nano-walls. Scale bars correspond to 500 nm in (**a**–**d**). 100 nm in (**e**) and 10 nm in (**f**).

Figure 2a shows a top view image of bare GNWs grown on SS. The nano-walls are homogeneously distributed and present an open structure with voids between the nano-walls. Their length is of several hundreds of nanometers and a few nanometer thick (about 10 graphene layers, see Figure 2e,f).

After deposition of MnO_2_, a layer of the oxide is observed coating the nano-walls. The presence of manganese and oxygen was determined by EDX (energy dispersive X-ray spectroscopy) and XPS [29] (see Figures S2 and S3 in supplementary information for more details). For samples without thermal treatment and annealed at 200 °C or below (Figure 2b,c, respectively), the open structure of the nano-walls is still recognized. However, a completely different morphology is presented by the sample annealed at 600 °C (Figure 2d) with a homogeneous layer of the oxide covering the nano-walls without visible voids and with a more compact structure.

**Figure 3:**
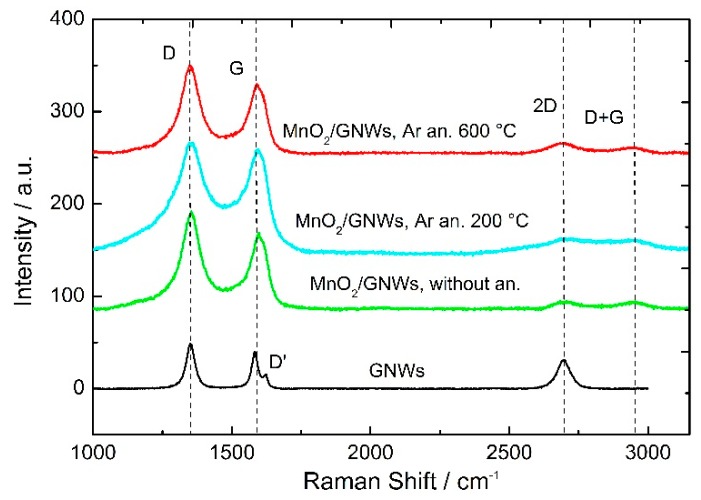
Raman spectra of GNWs grown on SS and MnO_2_/GNWs samples without annealing and Argon annealing at 200 and 600 °C.

Raman spectra of bare GNWs show typical D and G bands at 1346 cm^−1^ and 1587 cm^−1^ (Figure 3). The large number of defects (high D-peak) is associated with the plasma deposition technique. A shoulder appears next to the G band at 1616 cm^−1^ (D’ band) related to the finite size of graphite crystals and graphene edges [33]. Furthermore, the presence of a peak at 2700 cm^−1^ (2D) and I_2D_/I_G_ = 0.8 confirms the presence of few-layered graphene in accordance with TEM images (Figure 2e,f). An additional peak appears at 2940 cm^−1^ related to sp^2^ CH stretching vibrations [34]. MnO_2_/GNWs samples without and with annealing at different temperatures show similar Raman spectra, which indicate little or no alteration of the graphene nano-walls structure after MnO_2_ deposition and thermal treatment (Figure 3). Main D and G bands are clearly recognized for all the samples without big differences between them. However, unlike the bare GNWs sample, the D’ shoulder is not appreciable for these samples. In addition, 2D and D + G bands appear much weaker than for bare GNWs sample due to the presence of MnO_2_ coating on the surface and edges of the nano-walls. No specific bands of MnO_2_ are recorded, likely due to its low loading, low crystallinity, and/or small crystal size [30].

### 3.2. Electrochemical Performance 

The average specific capacitance of the samples was obtained from the cyclic voltammograms applying Equation (2).

(2) $$C_{s}=\frac{q_{a}+\left|q_{c}\right|}{2m\Delta V}$$

where C_s_ is the average specific capacitance in F·g^−1^, q_a_ and q_c_ are the anodic and cathodic charges, respectively in C. m is the mass of MnO_2_/GNWs composite in g and ∆V the applied voltage window in V.

Alternatively, the specific capacitance was also obtained from galvanostatic discharge curves, according to the following equation.

(3) $$C_{s}=\frac{I}{(\Delta V/\Delta t)m}$$

where C_s_ and m have the same meaning and units, as previously shown (Equation (2)). ∆V is the voltage difference during the discharge in V and ∆t the discharge time in s. I is the current applied during discharge in A.

**Figure 4:**
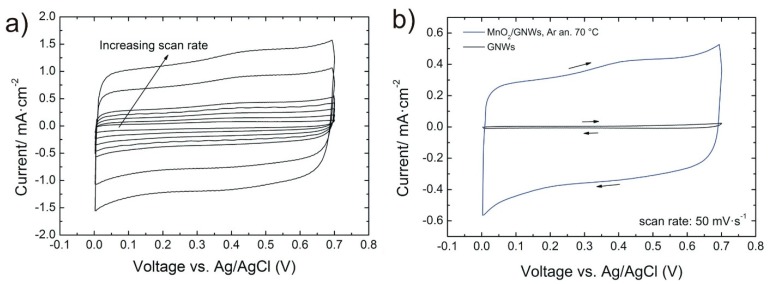
(**a**) Cyclic voltammograms of MnO_2_/GNWs sample annealed at 70 °C using scan rates 10, 20, 30, 40, 50, 100, and 150 mV·s^−1^. (**b**) Bare GNWs and MnO_2_/GNWs sample annealed at 70 °C using a scan rate of 50 mV·s^−1^ and a voltage window between 0.0 to 0.7 V vs. Ag/AgCl. Arrows indicate the direction of the scan.

Cyclic voltammograms of the samples exhibit a rectangular shape that indicates the capacitive nature of the GNWs and the MnO_2_/GNWs nanocomposites (Figure 4a,b). Deposition of MnO_2_ increases the current density of MnO_2_/GNWs by a factor greater than 20 compared to bare GNWs samples due to the pseudocapacitive effect of the transition metal oxide (Figure 4b).

**Figure 5:**
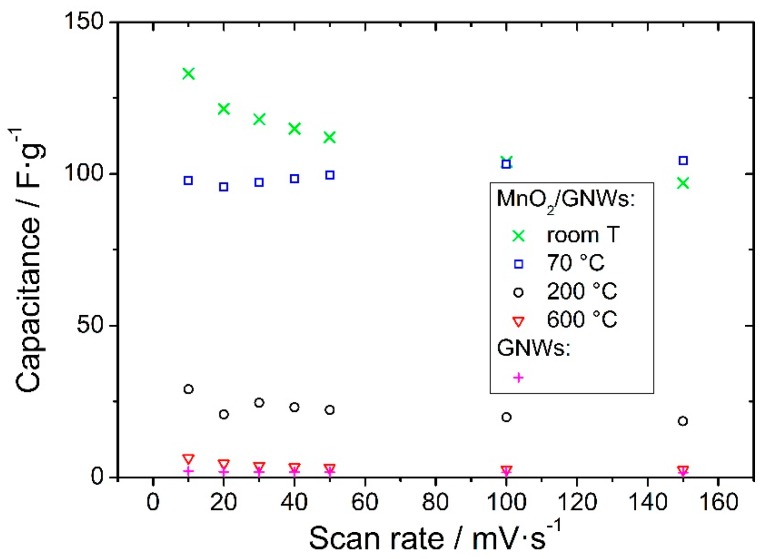
Specific capacitance of bare GNWs and MnO_2_/GNWs samples without (room T) and with annealing at different temperatures versus the scan rate.

**Figure 6:**
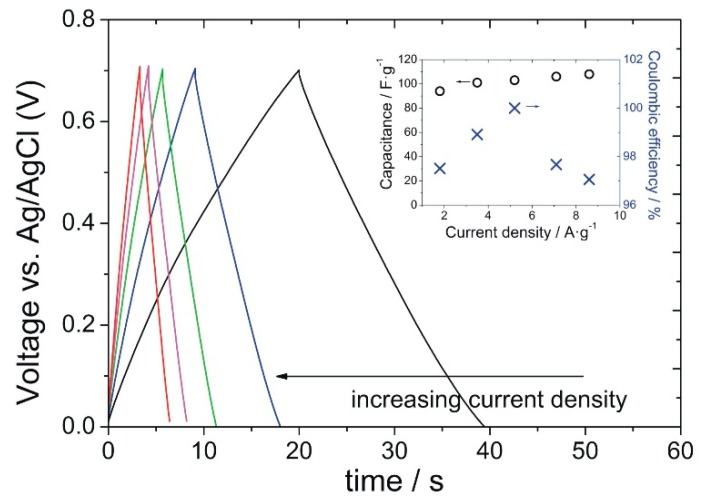
Galvanostatic charge/discharge curves of MnO_2_/GNWs sample annealed at 70 °C. The values obtained agree with those from CV measurements and show excellent coulombic efficiency (>97%) at high current densities (see inset graph), which were indicated by the symmetry of the charge/discharge curves.

**Figure 7:**
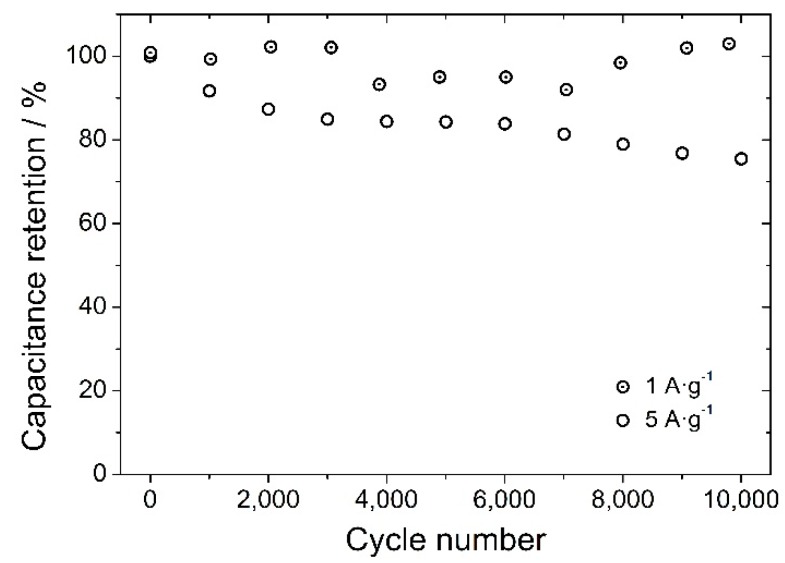
Cycling stability at different current densities (1 and 5 A·g^−1^) of MnO_2_/GNWs sample annealed at 70 °C. At 1 A·g^−1^, the capacitance retention remains almost constant and around 100% throughout 10k cycles. At 5 A·g^−1^, capacitance retention decreases down to 75% after 10k cycles and the coulombic efficiency is >98% throughout the whole measurement for both current densities.

At a scan rate of 10 mV·s^−1^, MnO_2_/GNWs sample without annealing provides the highest specific capacitance (SC) of 133 F·g^−1^ (Figure 5). However, at high scan rates (150 mV·s^−1^), the highest SC is presented by MnO_2_/GNWs sample annealed at 70 °C (104 F·g^−1^). Galvanostatic charge/discharge measurements are in agreement with these results. Figure 6 shows the galvanostatic charge/discharge curves for the sample annealed at 70 °C. The symmetrical triangular shape of the curves indicate excellent coulombic efficiency (>97%). The capacitance increases slightly with the current density (see inset graph in Figure 6), as observed from CV measurements (Figure 5). In addition, this sample also shows the highest cycling stability at high current densities (5 A·g^−1^) after 10,000 cycles and excellent cycling stability at low current densities (1 A·g^−1^) (Figure 7). The sample without thermal treatment shows a capacitance retention of only 50% at 3 A·g^−1^ after 10k cycles, and the rest of the samples present even poorer performance in comparison to the sample annealed at 70 °C.

### 3.3. Impedance Spectroscopy

Further information about the electrochemical processes taking place at the interfaces and bulk of the electrode materials can be obtained by means of electrochemical impedance spectroscopy.

**Figure 8:**
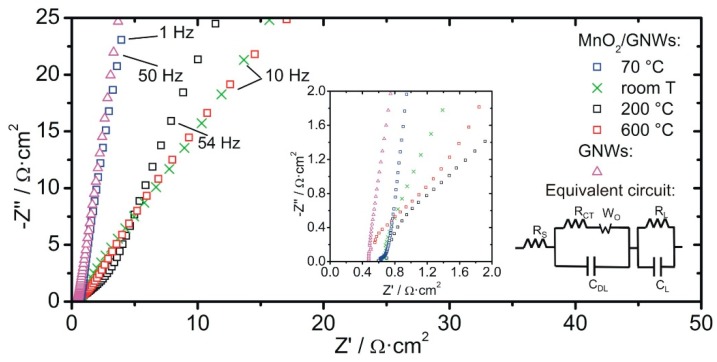
Nyquist plot of the samples: bare GNWs and MnO_2_/GNWs nanocomposite without annealing (room T) and annealed at 70 °C, 200 °C, and 600 °C. Inset shows real axis intercection and the modified Randles equivalent circuit used to fit the data of MnO_2_/GNWs.

The Nyquist plot of the samples is in accordance with their morphology and show the typical behavior for capacitive porous electrodes (Figure 8). A modified Randles circuit can be used to fit the experimental data of MnO_2_/GNWs [35] (Figure 8), which describe charge storage and transfer properties of the different interfaces and bulk of the electrode/electrolyte system. The equivalent circuit consist of capacitances (C) and resistances (R) in series and/or parallel configuration. The internal resistance of the cell is given by R_S_, which corresponds to the intersection point with the real axis at high frequencies in the Nyquist spectra (inset in Figure 8). The charge transfer resistance between the electrode and the electrolyte is represented by R_CT_. The double layer capacitance connected in parallel is given by C_DL_. In the mid-frequency region of the spectrum, a Warburg element (W_O_) describes diffusion processes of the ions through the porous structure of the electrodes, which is expressed as $A/{\left(j\omega \right)}^{n}$, where A is the Warburg coefficient, n is an exponent, and ω is the angular frequency. At low frequencies, the spectra show an almost straight line parallel to the imaginary axis, which is related to a perfect polarized capacitive behavior described by mass capacitance (C_L_), which is connected in parallel to a leakage resistance (R_L_) [36]. For the GNWs sample, a more simple circuit was used, which was composed of a resistance (R_S_) in series with a constant phase element describing an almost ideal capacitive behavior.

The whole spectra (1 Hz to 100 kHz) were fitted using ZVIEW software (Version 2.1, Scribner Associates, Inc., Southern Pines, NC, USA) and the equivalent circuit parameters obtained for MnO_2_/GNWs are shown in Table 1. GNWs presented a series resistance of 0.4 Ω·cm^2^ and a double layer capacitance of about 5 F·g^−1^, in accordance with CV measurements. 

**Figure 9:**
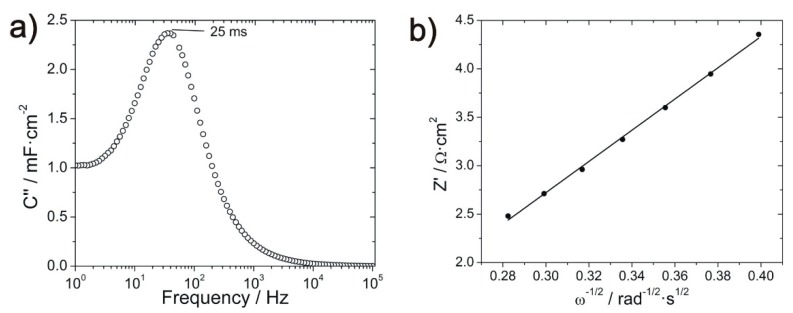
(**a**) C’’ vs. frequency plot to determine the relaxation time constant; (**b**) Z’ vs. ω^1/2^ plot to evaluate the diffusion coefficient of MnO_2_/GNWs heated at 70 °C.

Although ions from the bulk solution are transported throughout the porous electrode, it is known that proton diffusion inside the MnO_2_ lattice is a key step during charge/discharge [37]. Since the concentration of Na_2_SO_4_ is high, it is assumed that the diffusion of electrolyte ions through the porous electrode is fast in comparison to the diffusion of protons through the MnO_2_ lattice and, therefore, proton diffusion is the rate-determining step in the low frequency region. Thus, the proton diffusion coefficient (D) can be obtained from the slope of the Z’ vs. ω^1/2^ plot (Figure 9b) through the following equation [38,39].

(4) $$D=\frac{R^{2}T^{2}M^{2}}{{2A}^{2}n^{4}F^{4}\sigma^{2}}$$

where R is the gas constant in J·mol^−1^·K^−1^, T is the temperature in K, M is the molar volume (taken as 17.30 cm^3^·mol^−1^ for MnO_2_), A is the geometric area of the electrode in cm^2^, n is the number of electrons involved in the reaction, F is the Faraday constant in C·mol^−1^, and σ is the slope of Figure 9b in Ω·s^−1/2^. The maximum proton diffusion coefficient is obtained for the sample annealed at 70 °C with a value of 1.3 × 10^−13^ cm^2^·s^−1^.

The relaxation time constant (τ_0_) can be obtained from the C’’ vs. frequency plot (Figure 9a) using Equation (5) [36,40].

(5) $$C^{\prime \prime }=\frac{Z^{\prime }}{2\pi f{\left|Z\right|}^{2}}$$

where C’’ is the imaginary part of the capacitance in F·cm^2^, Z’ is the real part of the impedance in Ω·cm^2^, f is the frequency in Hz, and |Z| is the modulus of the impedance in Ω·cm^2^. The position of the maximum in Figure 9a provides the characteristic frequency, which is the inverse of τ_0_. The fastest relaxation time was 25 ms, which is obtained for the sample annealed at 70 °C.

## 4. Discussion

The heat-treated samples are expected to show lower open porosity and lower surface area, especially those heated at high temperatures above 200 °C. In addition, it has been suggested that high scan rates limits proton and Na^+^ ion diffusion and makes some pores and voids inaccessible [41], which implies a decrease in SC with a scan rate. It is also known that a heat treatment below 220 °C removes water and trace amounts of oxygen [42], and, around 500 °C, a transition occurs from α-MnO_2_ to crystalline Mn_2_O_3_ by loss of oxygen (chemical transformation) [42,43,44], which explains the low SC of the samples treated above 200 °C. However, the sample annealed at 70 °C shows a particular behavior increasing its SC with the scan rate (Figure 5 and inset graph in Figure 6). This phenomenon has already been reported previously and has been assigned to an excellent contact between the porous collector (GNWs in this case) and the oxide that allows efficient access of both electrons and ions to afford a fast redox reaction at high scan rates [45]. This redox process takes place at the surface of MnO_2_ where only a thin layer of MnO_2_ is involved and is electrochemically active [46]. 

Clearly, high annealing temperatures drastically reduce the capacitance due to the removal of bound water and lower open porosity of the electrode [41]. A mild treatment at 70 °C improves the SC and provides a better cycling stability even at high current densities (Figure 7). The Coulombic efficiency for this sample remained above 98% even after 10 k cycles at 1 and 5 A g^−1^, and the capacitance retention was excellent at 1 A·g^−1^ and 75% at 5 A·g^−1^.

The cycling stability of manganese oxides is mainly controlled by their microstructure (physical property), while their specific capacitance is governed primarily by their chemically hydrous state [47]. Thus, when the annealing temperature is too high (above 200 °C), the hydrous state is reduced and, without annealing or at low temperatures, the microstructure is not as stable as after a heat treatment. Optimum conditions are found at 70 °C annealing temperature.

As expected, the series resistance values are similar to all the samples, which are related to the electrolyte resistance, cables, and contact resistances of the cell [12] (Table 1). Due to the low conductivity of MnO_2_, the R_S_ value increases slightly after its deposition. However, after heating the sample at 600 °C, this value decreases again, probably due to detachment of the oxide layer from the GNWs exposing the nanocarbon material to the electrolyte. The charge transfer resistance presents its lowest value after annealing the MnO_2_/GNWs at 70 °C. It is assumed that this mild temperature allows better contact of the oxide with the GNWs and avoids fast water evaporation that can result in big voids, cracks, and bad adhesion between oxide and GNWs. This is probably the case for the sample annealed at 200 °C. At higher temperatures, the R_CT_ decreases again due to the increased diffusivity of the metal-oxide ions during annealing that result in better contact between GNWs and MnO_2_. Both double layer capacitance and mass capacitance have its maximum after annealing at 70 °C, which correlates with the cyclic voltammetry results. The leakage resistance diminishes with an increasing annealing temperature. The origin of this resistance is usually related to some faradaic process [22], and may be related to chemical reactions or phase changes of the manganese oxide. At annealing temperatures below 500 °C, there is a loss of water and a slight loss of oxygen [48], while a phase change from α-MnO_2_ to Mn_2_O_3_ takes place above 500 °C [47]. Lower resistance implies higher leaking currents and poor performance. Hence, low annealing temperatures should be used. The Warburg coefficient presents its lowest value for the sample annealed at 70 °C. The exponent n is usually 0.5 for porous carbon electrodes. The high value of 0.65 obtained for the 70 °C annealed sample signifies that ion diffusion takes place only at the surface of the electrode and does not behave as a typical porous electrode [35]. This result is in accordance with the proton diffusion coefficient (D) obtained from the slope of the Z’ vs. ω^1/2^ plot (Figure 9a).

In agreement with previously reported values [37], the obtained proton diffusion coefficients in manganese dioxide range from 3.3·× 10^−16^ to 2.4·× 10^−15^ cm^2^·s^−1^ for all the samples except for the one annealed at 70 °C, which presents a value of 1.3·× 10^−13^ cm^2^·s^−1^. The fast diffusion of protons in this sample is in accordance with the low Warburg coefficient and high n value obtained (Table 1).

The relaxation time is related to the power delivery and, ideally, should be as small as possible. The shortest relaxation time is obtained after annealing at 70 °C (Figure 9a), which facilitates fast rate performance during charge/discharge cycles.

Hassan et al. [21] also measured the capacitive properties of MnO_2_/carbon nano-walls (CNWs) composite on nickel foam, and obtained an SC of about 310 F·g^−1^ at 150 mV·s^−1^ considering the mass of MnO_2_ only. In this work, we obtained an SC of 278 F·g^−1^ (considering the mass of MnO_2_ only) at 150 mV·s^−1^ for the sample annealed at 70 °C. The two values are similar, but, in this case, we used SS as a substrate, which is more cost-effective.

In summary, graphene nano-walls have been successfully grown on conductive and flexible stainless steel substrate by ICP-CVD method. Galvanostatic electrodeposition of MnO_2_ increases the SC of the samples up to 133 F·g^−1^ at 10 mV·s^−1^ for the sample without thermal treatment. However, after a mild annealing at 70 °C, the MnO_2_/GNWs nanocomposite exhibits optimum electrochemical performance, i.e., small charge transfer resistance, high cycling stability, excellent coulombic efficiency, and SC even at high charge/discharge rates, which is related to optimum contact between GNWs and MnO_2_ and fast surface redox reactions. In accordance with these results, a maximum in the proton diffusion coefficient and short relaxation time is found at 70 °C annealing temperature, which suggests fast surface diffusion processes. Samples annealed above 200 °C show low SC and stability due to the reduced open porosity, bound water removal from manganese oxide, and faradaic reactions or phase changes.

## Figures and Tables

**Table 1:** Equivalent circuit parameters obtained from fitting the EIS data of MnO_2_/GNWs nanocomposites.

Electrode Materal/ Annealing T (°C)	R_S_ (Ω·cm^2^)	R_CT_ (Ω·cm^2^)	W_0_ = A/(jω)^n^ A (Ω·s^−n^) n		C_DL_ (F·g^−1^)	R_L_ (Ω·cm^2^)	C_L_ (F·g^−1^)
MnO_2_/GNWs/-	0.7	0.9	490	0.54	6.7	637	206
MnO_2_/GNWs/70	0.6	0.1	8.52	0.65	25	195	231
MnO_2_/GNWs/200	0.7	1.5	101	0.60	0.63	124	14
MnO_2_/GNWs/600	0.5	0.6	923	0.61	0.84	4.3	187

## Supplementary Materials

The following are available online at http://www.mdpi.com/1996-1944/12/3/483/s1, Figure S1: Calibration curve for the determination of manganese dioxide in the samples. The absorbance maximum at 525 nm is plotted against the concentration of MnO_4_^−^ in aqueous solution, Figure S2: Energy dispersive x-ray spectrum of a MnO2/GNWs composite sample after galvanostatic electrodeposition of MnO_2_, Figure S3: XPS spectra of galvanostatically electrodeposited MnO_2_ without thermal treatment.
