# Supplementary material for: Super-Capacitive Performance of Manganese Dioxide/Graphene Nano-Walls Electrodes Deposited on Stainless Steel Current Collectors
Source: Materials (Basel). 2019 Feb 4;12(3):483. doi: 10.3390/ma12030483 (PMC6390200; doi:10.3390/ma12030483)
Supplement: Supplementary file 1 [file materials-12-00483-s001.pdf]

materials

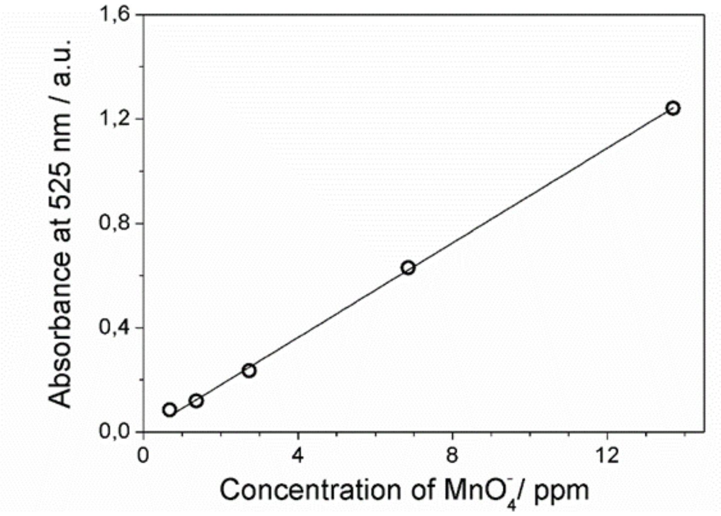

Figure S1. Calibration curve for the determination of manganese dioxide in the samples. The absorbance maximum at 525 nm is plotted against the concentration of MnO_4^- in aqueous solution.

MnO_4^- has a characteristic absorbance maximum at 525 nm, which can be used to qualitatively determine the mass loading of MnO_2 using UV–vis spectrophotometry [32]. The process was as follows: first the sample with electrodeposited MnO_2 is immersed into 10 mL of concentrated nitric acid to dissolve the oxide; then, the manganese ions are oxidized to MnO_4^- using a definite amount of $K_2S_2O_8$ and $AgNO_3$; the concentration of MnO_4^- ions is determined by UV–vis spectrophotometry (using Figure S1 as the calibration curve); finally, the loading mass of MnO_2 is calculated based on the concentration of MnO_4^- and the volume of the solution.

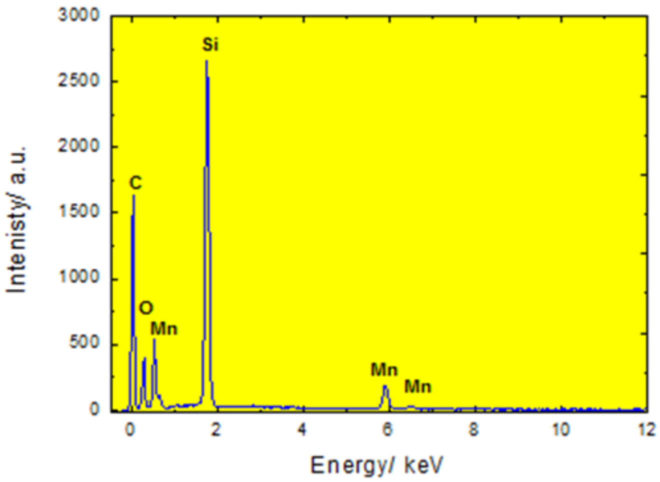

Figure S2. Energy dispersive x-ray spectrum of a MnO_2/GNWs composite sample after galvanostatic electrodeposition of MnO_2.

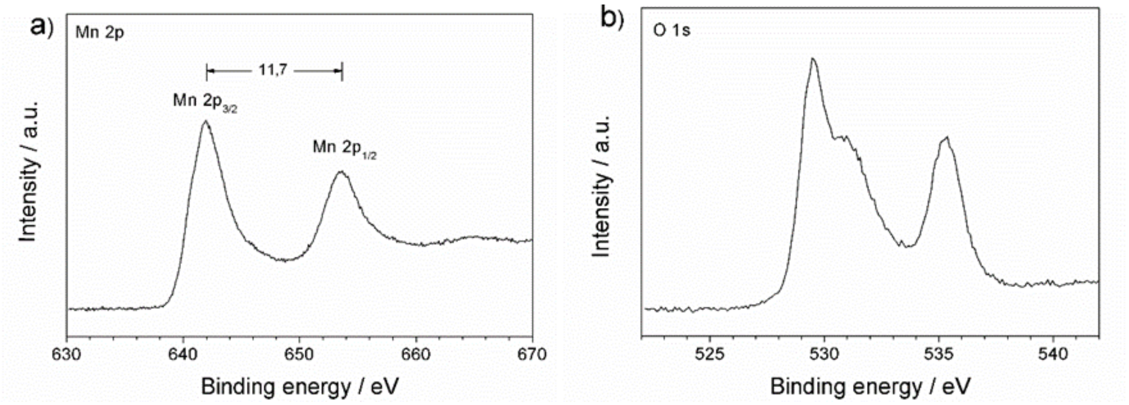

Figure S3. XPS spectra of galvanostatically electrodeposited MnO_2 without thermal treatment [33].

XPS was used to evaluate the elemental composition and oxidation state of manganese. Figure S3a shows Mn 2p spectra of galvanostatically electrodeposited MnO_2 without thermal treatment. The Mn 2p spectrum exhibits three peaks; Mn $2p_{3/2}$ was positioned at (641.85 ± 0.1) eV, Mn $2p_{1/2}$ at (653.5 ± 0.1) eV [1], and a shoulder appears at around 644.5 eV. The binding energy difference between the Mn $2p_{3/2}$ and Mn $2p_{1/2}$ peaks is about 11.7 eV which is very much like the reported values in other studies [2]. These results indicate that manganese is in the Mn^{4+} oxidation state [3].

Deconvolution of the O1s spectra in 4 peaks (see Figure S3b) reveals the chemical environment of oxygen. The peak located at (529.30 ± 0.1) eV can be ascribed to (Mn–O–Mn). Peaks around (530.9 ± 0.1) eV, (532.7 ± 0.2) eV, and (535.10 ± 0.2) eV could be attributed to (Mn–O–H), (H–O–H), and chemisorbed oxygen, respectively [4,5]. The position and percentage area of the deconvoluted O1s peaks were 529.44 eV/33.79%, 530.88 eV/24.36%, 532.39 eV/13.89%, 535.26 eV/27.96%, which correspond to Mn–O–Mn, Mn–O–OH, H–O–H and chemisorbed oxygen, respectively.

References

1. Sellers, M.C.K.; Castle, B.M.; Marsh, C.P. Threedimensional manganese dioxide-functionalized carbon nanotube electrodes for electrochemical supercapacitors. *J. Solid State Electr*. **2013,** *17*, 175–182.
2. Shen, J.; Liu, A.; Tu, Y. Asymmetric deposition of manganese oxide in single walled carbon nanotube films as electrodes for flexible high frequency response electrochemical capacitors. *Electrochim. Acta* **2012,** *78*, 122–132.
3. Nesbitt, H.W.; Banerjee, D. Interpretation of XPS Mn(2p) spectra of Mn oxyhydroxides and constraints on the mechanism ofMnO2 precipitation. *Am. Mineral.* **1998**, *83*, 305–315.
4. Dubal, D.P.; Dhawale, D.S.; Salunkhe, R.R.; Lokhande, C.D. Conversion of chemically prepared interlocked cubelike Mn3O4 to birnessite MnO2 using electrochemical cycling. *J. Electrochem. Soc*. **2010**, *157*, A812–A817.
5. Biniak, S.; Szymánski, G.; Siedlewski, J.; Swiatkoski, A. The characterization of activated carbons with oxygen and nitrogen surface groups. *Carbon* **1997**, *35*, 1799–1810.
